# Career change teachers in hard-to-staff schools: should I stay or leave?

**DOI:** 10.1007/s13384-023-00609-9

**Published:** 2023-02-13

**Authors:** Babak Dadvand, Jan van Driel, Chris Speldewinde, Merryn Dawborn-Gundlach

**Affiliations:** 1 School of Education, La Trobe University, ED1 Building, Level 5, Room 518, Bundoora, VIC 3086 Australia; 2 Melbourne Graduate School of Education, The University of Melbourne, 100 Leicester Street, Room L.411, Parkville, VIC 3010 Australia; 3 Melbourne Graduate School of Education, The University of Melbourne, Level 4, 100 Leicester St., Parkville, VIC 3010 Australia; 4 Melbourne Graduate School of Education, The University of Melbourne, Level 4, 100 Leicester Street, Carlton, VIC 3010 Australia

**Keywords:** Career change teachers, Career transition, Hard-to-staff schools, Initial Teacher Education, Teacher shortage, Teacher support

## Abstract

Recruiting career changers into teaching has emerged as a part of a strategy by governments worldwide to address complex teacher shortage problems in hard-to-staff schools. In this paper, we present a case study of two career change teachers and trace their career journey into Initial Teacher Education (ITE) and the teaching profession in two separate hard-to-staff schools. We interviewed these teachers during the first 2 years of their career change journey. During this period, ‘push-and-pull’ factors impacted their intentions to stay in the profession. Challenges included inadequate school-level mentorship support, social-geographic isolation in a regional school setting during the COVID-19 remote learning and the more complex working conditions in hard-to-staff schools. The adverse impacts of these challenges were, to some extent, mitigated by the participants’ commitment to making a positive difference in the lives of children and young people through the teaching profession, a strong work ethic and support provided by their ITE programme in the form of university-based mentors and adjustment to study requirements. The participants responded to these push-and-pull factors in ways that highlighted their reflexive decision-making and determination to stay in teaching despite challenges. We discuss the implications of these findings for workforce planning strategies aimed at recruiting career change teachers in hard-to-staff schools.

## Introduction

Interviewer: Have you considered leaving the teaching profession?

Maria: Oh yeah, of course!

Interviewer: Why?

Maria: I think the stress-workload combination. [I ask] why am I doing this to myself, which is a really legitimate question and one I’m actually really comfortable doing. It is because I think it’s important to query. I think it’s really important to come back to one’s motivation… to keep doing something that’s difficult.

Maria is a career change teacher enrolled in an employment-based Initial Teacher Education (ITE) programme in Victoria, Australia. At the time of the interview, Maria was undertaking the first year of her ITE while working at 0.8 Full-Time Equivalent (FTE) in a special school with a high proportion of students with high needs. We take the above exchange as a point of entry into a discussion about the career journey of individuals with professional backgrounds outside education into ITE and teaching in ‘hard-to-staff’ schools. This paper aims to provide a more nuanced understanding of how career change teachers navigate challenges in these schools in their early years of teaching, a critical make-or-break period in teacher retention (Australian Institute for Teaching & School Leadership, 2019). In doing so, we contribute to current debates about more effective policy responses to addressing teacher shortage problems in schools considered hard-to-staff due to one or a combination of factors relating to their student needs, socio-economic status (SES) or geographical location.

The number of career changers in teaching has been steadily growing. Yet, research remains in short supply as to how this growing cadre of teachers, many of whom enter the profession through employment-based teacher education pathways, navigate changing role expectations and shifting identities (Thomas & Mockler, 2018). In our research, we addressed part of this gap by examining the transition experiences of two career change teachers during critical walking point moments in their early years of teaching in two separate hard-to-staff schools. Using a case study approach, we traced their transition journey, identified their challenges, and the supports that helped them respond to adversities. These teachers worked within and against the odds while interrogating their decision to stay in or leave teaching. We provide an account of each participant in their personal, professional and institutional context focussing on the factors that mediated their career intentions. Our overarching aim is to contribute to debates about more coherent and effective policy solutions to chronic teacher shortages in hard-to-staff Australian schools.

## Career changers and chronic teacher shortages

Terms such as career change, mid-career or second career teachers are often used to refer to people who enter teaching from other professions (Haim & Amdur, 2016). Age has traditionally characterised career change teachers. For instance, Brandes’ (1985) earlier work suggests that career change decisions are more likely to occur around the age of 40 when individuals face a decline in career satisfaction. This ‘mid-life transition’ often sees individuals consider teaching an alternative career (Wilkins, 2017). Given the significant social and economic transformations of the past couple of decades, the profile of the career changers has also changed. This is reflected in more recent studies, which have defined career change teachers in broader terms as people over the age of 25 who have acquired significant life experience from previous occupations allowing them to bring assets such as maturity in age and professional expertise (Bar-Tal et al., 2020).

In this article, we use ‘career change teachers’ for those who enter teaching from other career backgrounds regardless of their age (Beutel et al., 2019). Internationally, career change teachers are increasingly viewed as part of ‘the solution’ to complex teacher shortage problems (Bauer et al., 2017). The Organisations for Economic Cooperation and Development (OECD), for instance, has called on governments to provide ‘alternative routes into teaching for mid-career changers that combine formal study and on-the-job support’ (OECD, 2005, p. 133). In recent years, there have been renewed calls to attract individuals from other fields and professions into teaching, primarily due to a shortage of qualified teachers in ‘high-demand’ subject areas such as Science, Technology, Engineering and Mathematics (European Commission, 2019).

The Australian Government Department of Education’s modelling shows that between 2021 and 2025, the demand for secondary school teachers will exceed the number of teacher graduates by approximately 4100 teachers (Department of Education, 2022). However, it is worth noting that the shortage of qualified teachers is not evenly distributed among schools. Schools that serve low socio-economic status (SES) students are about six times more likely to report teacher shortages compared to their more affluent counterparts (OECD, 2018). This has prompted various Australian governments to seek solutions such as fast-tracked entry pathways into teaching, relocation grants and financial incentives for working in hard-to-staff schools. Career change teachers are a part of the wider strategy to tackle existing and projected teacher shortages. At the national level, the Federal government’s Quality Initial Teacher Education Review has renewed calls to attract ‘high-quality’ mid-career changers into teaching (Department of Education Skills & Employment, 2022). In Victoria, where we undertook this research, some of the most recent State government initiatives have focussed on recruiting career change individuals as a lever to mitigate teacher shortages in hard-to-staff secondary schools (Victorian Government, 2022).

Despite increased attention to teacher shortages in hard-to-staff school settings, the dominant response to the problem has been through a series of disparate initiatives. This includes recent changes to the funding of higher education to reduce the cost of university courses in high-demand professions such as teaching (Department of Education Skills and Employment, 2020b), increased funding for employment-based ITE programmes to attract mid-career individuals (Department of Education Skills and Employment, 2020a) and financial incentives for teachers in hard-to-staff schools in regional and rural settings (Premier of Victoria, 2019). Although such front-focussed solutions can lead to a temporary increase in the teacher supply pool by attracting individuals to the profession, they are unlikely to significantly impact the problem they seek to address.

It is important to recognise the limitations and caveats of front-end-focussed workforce planning strategies that prioritise attracting career changers to teach in the more challenging working conditions of hard-to-staff schools without providing adequate support to retain them in these schools. Without attention to teacher retention, the teacher shortage problems in hard-to-staff schools, especially in remote/regional or low socio-economic status settings, will likely persist due to the revolving door of teacher recruitment and teacher attrition. Given their often more complex working conditions, teaching in these schools can generate higher stress levels for teachers, which can, in turn, adversely impact their career intentions (Kelly & Northrop, 2015).

## Career changers in the teaching profession

The recent incentives to attract career change individuals reflect a broader recognition of the positive contributions they can make to the teaching profession. Trent and Gao (2009) use the phrase ‘boundary crossers’ to discuss what characterises career changers as a distinct cohort within the teaching workforce. This includes their professional trajectories, previous work experiences and skill sets. Career change teachers tend to demonstrate a high capacity for workplace learning (Berger et al., 2012). They bring a repertoire of skills, including problem-solving skills, which allows them to respond to unpredictable circumstances (Bar-Tal & Gilat, 2019). The ability to problem-solve is often complemented by the experiences of working in the labour market, which can help make learning less abstract for students (Crosswell & Beutel, 2017). For instance, research has shown that science professionals in teaching can make science learning more tangible for their students because of their practical dispositions (Antink-Meyer & Brown, 2017).

Career change decisions can be motivated by a combination of altruistic, personal and labour-market-related factors. Altruistic factors include a strong sense of ‘calling for service’ (King, 2008), commitment to care for others (Williams & Forgasz, 2009) and a desire to make a difference in the education of children and young people (Bauer et al., 2017). Often intrinsically motivated, career change teachers bring strong work ethics and an intent to serve the common good (Tigchelaar et al., 2010). Personal factors include life–work balance or life-changing circumstances such as childbearing and care responsibilities in the family (Wagner & Imanel-Noy, 2014). Labour market factors are related to employment conditions, which can facilitate individuals’ decisions to change careers. Labour market-related factors are believed to contribute to a higher supply of teachers during times of economic downturn and recessions (See & Gorard, 2019).

Entering teaching as a career changer, however, is not without challenges. Changing careers can create financial distress due to a decrease in or a temporary loss of income for those with more established career backgrounds (Lee, 2011). Financial responsibility and caring duties towards family while attending to studies and meeting professional requirements during the early years of teaching can also create friction in the transition period (Bar-Tal & Biberman-Shalev, 2022; Varadharajan et al., 2020). While the initial forays into schools can present the excitement of a fresh start, experiences can change quickly to ‘reality shock’ once career change teachers are confronted with the day-to-day teaching requirements and associated administration (Crosswell & Beutel, 2017). This can be compounded by misconceptions about how easily one can transfer knowledge and skills from the previous profession into teaching (Troesch & Bauer, 2017).

The notion of ‘praxis shock’ can be particularly relevant to the early years’ experiences of career change teachers. Praxis shock happens when teachers confront the ‘realities and responsibilities of being a classroom teacher that puts their beliefs and ideas about teaching to the test, challenges some of them, and confirms others’ (Kelchtermans & Ballet, 2002, p. 105). For many career change teachers, an initial expectation for a fulfilling career experience can clash with what transpires in a classroom, including managing student behaviour, using effective teaching strategies, or addressing the additional needs of students. More often than not, the skills from a previous career may not be replicable directly in a classroom setting (Trent & Gao, 2009).

The praxis shock can be compounded for career change teachers by the need to reposition their professional identity as a novice despite, at times, significant track records in previous occupations (Beutel et al., 2019). Among other contributors to praxis shock are the well-documented challenges of teaching, including heavy workloads, teaching out of the field, lack of autonomy and insecure employment (Gallant & Riley, 2017; Redding & Henry, 2019; Thomas et al., 2019). Despair might follow when teachers face challenges that contradict the values driving their career intention. Santoro’s (2018) notion of ‘demoralisation’ provides a powerful account of how tensions between one’s beliefs about ‘good teaching’ and unresponsive school policies and practices can dampen teacher morale and ultimately lead the way to their exit from the profession.

The challenges of teaching are more significant in hard-to-staff schools where teachers often have access to limited resources (Allen et al., 2018). Despite efforts to address teacher shortages in these schools through recruiting career change teachers, we know little about how this cohort of the teaching workforce deals with change and challenges during their transition into ITE and teaching in schools considered as hard-to-staff. Addressing the underlying contributors to persistent and chronic teacher shortage problems in hard-to-staff schools requires careful consideration of the lived experience of the teachers in these schools and the support systems that can help retain them during the early years of teaching and beyond. Without such consideration, positioning career change teachers as the solution to complex and chronic teacher shortage problems in hard-to-staff schools will be inadequate, leading to a vicious cycle of teacher recruitment-attrition.

## The study

The data reported in this paper were collected as part of a broader study to understand the experiences of individuals who change careers to become teachers. The participants were enrolled in a 2-year employment-based ITE programme in the State of Victoria, Australia, that combined intensive study with teaching at 0.8 FTE (4 days a week) on an entry-level salary. In addition to investment from the Federal government (Department of Education Skills and Employment, 2020a), the Victorian government has also allocated significant funds to employment-based ITE programmes to offer flexible entry pathways, especially to career changers, as part of a wider solution to addressing teacher shortage problems in hard-to-staff schools (Premier of Victoria, 2019). We collected data from one such ITE programme using an announcement on the Learning Management System (LMS) inviting individuals with career experiences to participate in the research. Eighteen people responded to the open call for participation and were interviewed in October–November 2021. The sample included participants from two cohorts: those who started their ITE programme and teaching career in 2019 (second-year candidates) and those who began in 2020 (first-year candidates).

Once they expressed interest, the participants were formally invited via email with a Plain Language Statement (PLS) and Consent Form. Each participant then took part in a semi-structured interview that lasted between 30 and 45 min. These interviews probed into several questions, including the support provided by the university, managing academic studies with teaching commitments, and significant challenges faced in the adjustment to teaching. The interviews were conducted over Zoom and were audio-recorded for transcription before analysis. A thematic analysis approach was used to collate the transcribed data into segments based on similarities and differences (Merriam, 2009). The data segments represented emerging themes organised around common patterns relating to the diversity of the challenges the participants faced during their career transition into ITE and teaching and the support structures that helped them navigate challenges.

While the data identified commonalities in responses, differences also emerged in response patterns among the participants. This was most noticeable in answers to a question about future intention to stay in teaching. Most participants responded positively to this question, confirming their intent to remain in the job. However, two career change teachers teaching in separate hard-to-staff secondary schools indicated doubt and ambivalence in their responses. In this paper, we draw upon the data collected from these two participants. We take their stories as the case in point of two career change teachers grappling with doubt in the hard-to-staff schools where they began their teaching careers. Despite their doubt and ambivalence, these participants worked within and against the odds to stay in the profession. We maintain that valuable lessons can be learned from their experiences about how the push-and-pull factors impact career change teachers’ early years’ experiences and intentions. In our discussions, we refer to these two participants as Marcus and Maria (pseudonyms).

### Becoming a teacher: where the journey begins

Despite recognised challenges associated with teaching, a commitment to care and a desire to make a difference acted as a catalyst for the career change decision of Marcus and Maria, who had previous experiences across a range of ‘caring professions’ before deciding to pursue teaching. Marcus had a bachelor’s degree in Science and had worked in a hospital setting in medical record-keeping for a few years before his career change decision. As Marcus explained, he had always been interested in teaching, which he attributed partly to an intention to remain in and serve his regional hometown. Marcus went on to explain how this would have been more difficult to do had he worked in other fields given limited career opportunities in regional Victoria:

Marcus: I think teaching was always something I wanted to do on some level. My Mum’s a primary school teacher and I’d actually looked at the program I think a few years before when I was thinking about what is a [Bachelor of] Science degree is going to lead to… because being in a lab didn’t appeal to me and I think I wanted to stay rural and there’s limited opportunities to do science up here but teaching is something that I was interested in doing.

Maria had extensive experience in youth care before enrolling in her ITE programme. Having graduated as an Art Therapist about 20 years ago, Maria had worked with homeless youth and children with trauma experience. She had also worked for the Peace Corps, which led to her later relocation from the United States to Australia. Maria’s more recent professional experiences included working as a special needs advisor for the Department of Education in Samoa and as a school counsellor in an alternative school in Australia. This gave Maria, as she went on to explain, considerable experience in educational settings with learning and mental health and also furthered her passion for teaching, which ultimately paved the way for her career change decision:

Maria: I’ve sort of been intertwined in the profession my whole sort of working professional life. I’ve been in schools and classrooms. […] So basically, my journey has kind of moved all around and working a lot in the trauma sector across Australia in out of home care, of family violence, refugees and asylum seekers and then with Aboriginal organisations working with Aboriginal children and care.

A background in the caring sector was a commonality between Marcus’ and Maria’s career journey before becoming teachers. This highlights what Hansen (2021) refers to as the ethics of ‘calling to service’, which can act as a driver of individuals’ decision to become teachers. Despite their interest in teaching, a combination of circumstantial and pragmatic reasons, and an unwillingness to return to full-time study on campus, initially prevented Marcus and Maria from deciding to change careers. This changed with their enrolment in a 2-year employment-based pathway into teaching at the University of Melbourne. Both Marcus and Maria valued the flexibility of their ITE programme, which allowed them to pursue teaching while building their experiences and earning a salary. However, their relatively heavy teaching workload left them with one day per week to meet study requirements. This was not an easy undertaking. Marcus described his workload and study combination as ‘a beast’ requiring meticulous ‘time and energy management’:

Marcus: I felt I had to be very disciplined in that one day a week, the study day I had to really keep that sacred, as this is my day where I’m doing uni work. Sometimes, it was a day off because you know the energy balance gets a bit… it gets a bit hard but as long as I was able to do a piece of work, each week it was manageable and there were plenty of tasks I was doing in my teaching; they actually did apply themselves to the assignments and things.

Research has shown the positive impacts of support during ITE preparation on pre-service teachers’ preparedness (Keese et al., 2022). This was attested to by Marcus and Maria, who attributed significance to the support offered by their ITE provider. The ITE support ranged from providing what Marcus described as course content that was ‘applicable to teaching’ to opportunities to build ‘a support network’ with other colleagues enrolled in the same ITE programme during the university study intensive periods. For Maria, providing a university-based mentor with practical skills was deemed significant, especially in developing lesson plans and managing classrooms. Similarly, as Marcus explained, having a university-based mentor ‘independent from the school’, yet experienced in teaching and learning in school settings, gave him opportunities to seek additional feedback on his practice. This proved particularly valuable given what Marcus described as having to work in a ‘quite a small school’ where things have, for long, been ‘done the same way’.

### Working within and against the grain

Among the 17 career change teachers, we interviewed (Dadvand et al., 2022), Marcus’ and Maria’s accounts stood out as they questioned their decision to continue their initial teacher education course, although they had made an initial conscious decision to become teachers. While grappling with doubt and ambivalence, Marcus and Maria found ways to stay in the profession despite the challenges they face in working in two different hard-to-staff school settings. This section examines some of the tensions that led Marcus to move to a different school at the end of his second year of study and Maria to change her ITE course at the end of her first year to a standard pathway that did not require her to undertake a concurrent teaching workload. We examine how Marcus and Maria navigated challenges at a critical period of their transition into ITE and discuss how push-and-pull factors impacted their career experiences, intentions and decisions.

Many of the career change teachers we interviewed as part of our broader study valued the school-level mentor support they received. However, Marcus’ and Maria’s experiences with their school mentors ranged from mixed to inadequate. Located in a regional town, the school where Marcus commenced his teaching career had a high concentration of students from low SES backgrounds. The 2021 Index of Community Socio-Economic Advantage (ICSEA)^1^ from the My School Website^2^ showed that about half of the students in the school were from the bottom quarter of ICSEA distribution. In the interview, Marcus talked about having been assigned a formal school mentor who provided him with a point of contact for everyday queries regarding teaching and school duties. This approach to mentorship, however, proved insufficient due to its loose structure and reactive nature. Marcus went on to explain that despite being a ‘relatively independent person’, such an unstructured approach to mentorship support meant that he had to navigate challenges primarily on his own and only revert to his school mentor when things escalated:

Marcus: Probably at the school the ability to kind of support in terms of having rigorous systems and procedures in place for dealing with things probably wasn’t all there. I think during first term there were a few incidents where I didn’t necessarily feel all that supported, and that was when I sort of started to think, maybe I can’t be stuffed doing this, particularly for much longer. […] The school provided… so there was a mentor, so that was a teacher who [I] was assigned to and I actually sat next to my mentor in my first year… so their desk was next to mine which was useful in terms of you know how to use the photocopier, how do I do this, what am I supposed to do when they put me on yard duty, you know… all that kind of stuff. My first year I probably didn’t have much. I tend to be fairly independent anyways, so help was always kind of there if I needed it, but not necessarily like a structured kind of checking in type things, so I think I tended to kind of work things out and then sort of asked my mentor if I needed to know how to do something.

Marcus’ lack of initial experience in teaching is part and parcel of the entry pathway into the profession that allowed him to change his career in the first place. Employment-based ITE programmes such as the one Marcus and Maria were enrolled in combine intensive university preparation with teaching duties from the onset of their ITE programme (either on a provisional teacher registration or as paraprofessionals). Despite the recent funding support that these programmes have received from the Victorian government to incentivise mid-career professionals into the teaching profession in hard-to-staff schools (Department of Education Skills & Employment, 2022), these pathways tend to offer limited opportunities to ‘preview’ the job before teaching as a registered teacher. This makes their cohort of the teaching workforce particularly sensitive to the social conditions of their schools and the professional support that they receive on the job as a considerable part of their ‘learning to teach’ coincides with teaching (semi)independently in schools (Redding & Henry, 2019).

Unlike Marcus, who recounted a mixed experience with mentorship support, Maria’s mentorship account could be described as ‘insufficient’. Maria expressed her frustration about being placed in a school that catered for students with a combination of abilities and challenges. Due to its small number of teaching staff, the school’s capacity to support Maria was significantly limited. While Maria was formally assigned to a mentor, the mentorship became increasingly ‘less beneficial’ for her over time, something she attributed to her professional background in therapy which, according to Maria, impacted her mentor–mentee dynamics by pivoting the mentorship to chiefly non-teaching matters. As Maria further explained, this put her in a rather precarious position of being viewed as a person with significant professional experience while at the same time having to navigate the uncertain terrain of ‘learning to teach’ in the first year of her school experience:

Maria: The first two weeks, of course, I was like shaky voice, freaked out, anxious in front of the students, even though I’ve been a therapist for yonks, and I’ve done heaps of like professional development, training for adults and even young people group work… it’s still different. You’re still a novice; you’re absolutely a novice standing in front, trying to do something, and you don’t know what you’re doing and you’re trying really hard. […] I was there, so with the mentor and probably the meetings became less beneficial for my development because of the unfolding and undoing of the working relationship stuff and because my husband and I joke that it says therapist across my forehead… that sometimes people end up just telling me stuff about themselves. I’m like, ‘yeah alright!’, thinking, ‘far out, this is my time, but got it!’

Maria’s predicament is common among other career change teachers who often grapple with paradoxes and tensions in their career change journey. In their study of the views and experiences of 508 career change teachers who were enrolled in ITE programmes in Australia, Varadharajan et al. (2018), for instance, found such paradoxes and tensions can emanate from a range of factors, including being highly skilled and experienced on the one hand and inhabiting the status of ‘student–teacher’ in need of support on the other hand. These tensions position career change teachers, according to Varadharajan et al. (2018, pp. 741–742), as ‘experienced-neophytes’, that is, having ‘considerable life and/or work experience and a commensurate capacity for autonomy and independence [yet] armed only with their student perspective of learning, and as such, are quite dependent, in terms of acquiring new knowledge and a new teacher perspective’.

The paradoxes and tensions that Maria encountered were compounded due to working in a hard-to-staff school that catered for diverse students, including gifted students and those with disabilities. The school choice for Maria was informed by her previous professional background, which was deemed highly relevant to the requirement of working in such a setting. Maria’s work experience with ‘at risk’ and ‘marginalised’ youth in her previous professions prepared her, to some extent, to deal with a spectrum of mental health, disability and well-being issues in her day-to-day schoolwork. However, the added responsibility of managing classrooms, developing lesson plans, teaching and ensuring student learning heightened the professional challenges that she experienced. As a case in point of these additional challenges, Maria recounted how she was challenged in a classroom encounter involving a distressing situation with a student who had complex needs:

Maria: There was an incident where you know […] one [student] had kind of laid on the floor and wrapped this kind of hose coil thing around his neck and was kind of refusing to not… I was engaging, backing off, engaging in because he also has a diagnosis of autism, ADHD and PDA. So, there’s this back forth space, checking in back; that goes on it with someone lying on the floor that’s about the same size as you, saying ‘I’m going to kill myself’.

Marcus’ first year of teaching was also marked with challenges, albeit of different sorts and nature. Coming from what he described as ‘a small town’, Marcus started teaching in a school located in a regional town outside Melbourne metropolitan area. Living and working in a school in a very small regional town created a sense of social and geographical isolation for Marcus. School closures caused by the COVID-19 pandemic and the subsequent transition to online teaching exacerbated Marcus’ solitary experience in the town. In response to a question about if he had ever re-considered his decision to stay in the teaching profession, Marcus lamented how a feeling of working in isolation in the first 2 years of his teaching career coupled with insufficient school-level support led him to re-think his decision and future intention to stay:

Marcus: It’s maintaining social connections and also support networks; it’s a stressful job, particularly if you don’t have everything going good outside of school as well, and you know, being able to go out and do things on the weekend and all the things we miss. That was also probably… the challenges became more external probably once you stop you know… having a focus so hard on, ‘right, what am I doing today? What am I doing tomorrow?’ Those other things start to happen.

In response to these challenges, Marcus ultimately decided ‘to look elsewhere and find a different school’. He was able to move to a different school in his hometown. The new school offered Marcus what he deemed largely missing from his previous school experience, namely adequate support. Marcus explained enthusiastically how he was teaching ‘a bit of everything’ including history, digital technologies, science and mathematics in his new school. Despite teaching out of his areas of expertise, Marcus found his new position ‘intellectually challenging’ as it prompted him to extend his acquired teaching skills to other learning areas. For Maria, however, the challenges in the first year of study and teaching led to a reconsideration of her entry pathway into teaching, ultimately paving the way for a change from the employment-based ITE programme to a standard pathway into teaching that did not combine the additional requirement of teaching with the study.

## Discussion and conclusion

Marcus’ and Maria’s journey into teaching can be emblematic of other career changers who may find themselves grappling with doubt and ambivalence as they navigate challenges when they enter teaching into hard-to-staff schools. In this latter section of the paper, we reflect on elements of Marcus’ and Maria’s career stories presented thus far to extrapolate two points that can have broader implications for research and workforce planning strategies that rely on career changers to solve education system-level problems relating to teacher shortages in these school settings. The first point that merits attention is how Maria and Marcus responded to inadequate support while working in challenges conditions. Far from denoting ‘an exit’, these responses indicated a process of reflexive decision-making to remain in the job. Marcus’ decision to change school and Maria’s decision to move to a standard ITE programme can be viewed as strategies that helped to ensure their sustainability within the profession. These strategic acts of decision-making, however, also highlight that the terms under which teachers decide to stay are not unconditional.

A contextual approach to understanding teacher resilience can help explain why Marcus and Maria retracted from unresponsive working conditions that ignored their career expectations and well-being. Such an approach recognises that ‘resilience lies at the interface of person and context, where individuals use strategies to enable them to overcome challenges and sustain their commitment and sense of wellbeing’ (Beltman, 2021, p. 15). Framing teacher resilience through a contextual lens helps acknowledge that the impetus to care for others, which drives the decision of individuals like Marcus and Maria to teach in hard-to staff schools, co-exists within a context of caring for the self. As Hansen (2021) has aptly pointed out, the idea of teaching as a calling to service, with its sense of commitment to making a difference, negates a wholesale form of self-abnegation on the part of teachers in the service of others. Our study provides further evidence of this, demonstrating how teachers act ethically yet strategically to retract from unsustainable conditions unresponsive to their needs and well-being while seeking viable alternatives to stay in the job.

The second point relates to the implications of our findings for the recruitment, preparation and retention of career changers for hard-to-staff schools. In the absence of adequate teacher support, initiatives that provide shortcuts to the profession as a way of addressing systemic teacher shortages in these school settings are unlikely to solve the problem they address. This is due to a combination of factors, including their often-fast-tracked ITE programmes (Carver-Thomas & Darling-Hammond, 2017) and the high-intensity working conditions of the schools where they teach. This makes these teachers heavily reliant on the social conditions and the support provided to them (Redding & Henry, 2019), further highlighting the important role that aspects of teacher preparation and school-based support play in boosting teacher morale and improving their retention (Keese et al., 2022). Given that teachers in hard-to-staff schools are likely to be less experienced and more strained in the Australian context (OECD, 2018), it is not surprising that (novice) teachers working in these schools may not receive adequate support, leaving them relatively under-prepared and demoralised.

We recognise the limitations and caveats of generalising from limited, albeit in-depth, data that we have reported to the broader cohort of career change teachers. However, our shared experiences of working with such cohorts as teacher educators and programme coordinator have led us to treat Marcus’ and Maria’s career narratives as a stark reminder of the need for a more cohesive workforce planning strategy for hard-to-staff schools that serve the most marginalised Australian communities. Caught up in a system of education administration marked by inadequate funding to tackle disadvantages (Gonski et al., 2011; Kenway, 2013), many of these schools, like the ones that Marcus and Maria left behind, end up in a vicious cycle in which they have to spend their limited resources to recruit new teachers, only to see them leave due to lack of support mechanisms before they develop the experience and skills to manage their role and contribute to student learning.

A more cohesive strategy for staffing hard-to-staff schools adopts a long-term agenda to address the well-recognised challenges that mark the teaching profession more broadly in Australia, such as excessive workloads, inadequate support, loss of professional trust and growing bureaucratisation of teaching (Heffernan et al., 2019). These challenges can act as a catalyst for demoralisation and lead to teacher exit decisions (Santoro, 2018). A less fragmented strategy also addresses the school-level conditions most conducive to teacher retention in hard-to-staff schools. School-level support for teachers like Maria and Marcus would not be possible without a significant reallocation of funds to better staff these schools and provide the resources to help retain their existing workforce. Such support provisions offer the new entrants into the teaching profession, including career changers, what they need to stay. In the cases of Marcus and Maria, this requires addressing the structural problems within the education system that generate teacher shortages in certain schools while attending to the cohort-specific needs of those who enter teaching from other career backgrounds.

## Footnotes

1 The Index of Community Socio-Educational Advantage (ICSEA) is a measure that provides information about the level of educational (dis)advantage of students and allows comparison of schools with similar student demographic on their performance over the National Assessment Programme: Literacy and Numeracy (NAPLAN).

2 My School Website is an online platform that offers publicly available information about schools, including information about school socio-demographics, student profiles as well as school performance over NAPLAN.
